# An efficient *in vitro*-inoculation method for *Tomato yellow leaf curl virus*

**DOI:** 10.1186/1743-422X-7-84

**Published:** 2010-04-29

**Authors:** Ayed M Al Abdallat, Hmoud S Al Debei, Heba Asmar, Samar Misbeh, Ayat Quraan, Anders Kvarnheden

**Affiliations:** 1 Department of Horticulture and Crop Science, Faculty of Agriculture, University of Jordan, Amman 11942, Jordan; 2 Agricultural Biotechnology Laboratories, Hamdi Mango Center for Scientific Research, University of Jordan, Amman 11942, Jordan; 3 Department of Plant Biology and Forest Genetics, Uppsala BioCenter SLU, Box 7080, SE-750 07 Uppsala, Sweden

## Abstract

**Background:**

*Tomato yellow leaf curl virus *(TYLCV) is a member of the family *Geminiviridae*, genus *Begomovirus*. To test the infectivity of TYLCV in tomato plants, an improved protocol for inoculation of *in vitro*-cultured tomato plants was developed.

**Results:**

A TYLCV isolate was cloned, sequenced and used to construct a 1.8-mer infectious clone. Three weeks old microshoots of TYLCV-susceptible tomato plants were inoculated with *Agrobacterium tumefaciens *harboring the infectious clone for the TYLCV isolate. After two weeks, the TYLCV symptoms started to appear on the *in vitro*-inoculated plants and the symptoms became more severe and pronounced eight weeks post-inoculation. The method was used efficiently to uncover the resistance mechanism against TYLCV in *Solanum habrochaites *accession LA 1777, a wild tomato known for its high resistance to whitefly and TYLCV.

**Conclusions:**

The reported *in vitro*-inoculation method can be used to screen tomato genotypes for their responses to TYLCV under controlled conditions and it will be a useful tool for better understanding of the TYLCV biology in tomato plants.

## Background

Since it was first reported in the Jordan valley [1], the tomato yellow leaf curl disease (TYLCD), caused by the *Tomato yellow leaf curl virus *(TYLCV) and related viruses, has become a serious problem that affects tomato (*Solanum lycopersicum*) worldwide. TYLCV is a member of the family *Geminiviridae*, genus *Begomovirus *and it is transmitted to tomato by the whitefly *Bemisia tabaci *in a persistent and circulative manner [2]. The management of the disease in tomato production areas is difficult and expensive and the control measurements are focused mainly on the whitefly control and are based on insecticide treatments and/or the use of physical barriers [3].

One of the best ways to reduce TYLCV damage is to breed plants resistant to both the virus and the vector. Breeding programs for TYLCV-resistant cultivars are based on the transfer of TYLCV resistance genes from wild tomato species into cultivated tomato [4]. Previous studies have revealed the presence of resistance mechanisms against the virus in wild tomato species that are controlled by multiple genes [4-6]. For instance, the TYLCV resistance in *S. chilense *is controlled by a major gene, termed *Ty-1*, and at least two other modifier genes [7]. However, the progress in the breeding program has been slow, primarily due to the complexity of TYLCV-resistance genetics and the virus-vector-host interactions [5]. The complexity of TYLCV resistance is reflected by the presence of different resistance mechanisms against the virus and the whitefly. In addition, TYLCD may be caused by different strains of TYLCV as well as other begomovirus species, such as *Tomato yellow leaf curl Sardinia virus *(TYLCSV).

To test the infectivity of TYLCV and to understand mechanisms of TYLCV resistance in plants, several methods for *in vivo *screening have been developed such as natural field infection, whitefly inoculation in cages, inoculation with the virus using leaf or stem agroinfiltration or biolistic inoculation [8]. In many instances, such methods have proven to be laborious and pose a potential threat to the environment. For instance, several susceptible tomato plants had escaped TYLCV infection using the method of natural field infection even 90 days after transplanting [9], while with whitefly inoculation in cages, it might be difficult to control the inoculum pressure [10]. In addition, such natural inoculation methods are not efficient for screening wild tomato species due to their non-preference by whiteflies. In addition, vector activity, virus multiplication and symptom development are affected by the prevalent environmental conditions during and after greenhouse or field inoculation. Agroinfiltration of stems or leaves are laborious, difficult and inefficient in some tomato genotypes [11,12]. Using biolistic inoculations, discrepancies in results related to plant species have also been reported [13-15]. Above all, testing for TYLCV resistance in transgenic plants using *in vivo *inoculation methods is difficult in open environment or non-specialized greenhouse conditions due to strict regulations for genetically modified organisms. Therefore, there is a need to establish controlled inoculation protocols to prevent any unfavorable spread of the viruses to the surrounding environment, especially when testing new viral strains or recombinants.

Two previous reports have described the development of viral inoculation systems suitable for *in vitro *plants [16,17]. With the described systems, it has been possible to inoculate successfully plants grown *in vitro *using a mechanical approach. In this study, we report the development and use of an efficient *in vitro *method suitable for TYLCV inoculation of tomato. The principle for the new inoculation method depends on dipping the basal part of the plant in a solution containing agrobacteria with an infectious TYLCV clone. The method was used successfully to inoculate susceptible tomato plants with TYLCV and to test for TYLCV resistance in wild tomato plants.

## Methods

### Cloning of a TYLCV genome

Leaves from a tomato plant showing TYLCD symptoms were collected from greenhouse-grown plants. Total DNA was extracted from the collected leaves using a CTAB method [18]. The overlapping primer method was used for the amplification of a full-length TYLCV DNA genome with the polymerase chain reaction (PCR) as described previously [19]. In this method, two designed primers, TYJU fwd (5'-TAAATA*CCATGG*CCGCGCAGCGGAATACACGACGTTC-3') and TYJU Rev (5'-TATAAT*CCATGG*AGACCCATAAGTATTGTCATTGAGGGTGA-3'), that overlap a conserved *Nco*I site (in italics) in the *C1 *gene of TYLCV were used in combination with the genomic DNA extract prepared from TYLCV-infected tomato leaf tissue in a PCR. The reactions were performed in a 25 μL volume containing 100 ng genomic DNA, 2.5 μL of dNTPs (100 μM), 5 μL of 5× PCR, 0.5 μM of each primer and 0.25 μL of 5 U/μL GoTaq DNA polymerase (Promega, Madison, Wisconsin). The PCR conditions were 94°C for 5 min, followed by 40 cycles of 94°C for 30 min, 55°C for 1 min, and 72°C for 1 min, and a final 10 min extension at 72°C. The PCR-amplified DNA fragments were digested with the restriction enzyme *Nco*I and the resulting ~2.8 kb DNA fragment was inserted into the same restriction site of the plasmid pCAMBIA1380 (Cambia, Canberra, Australia). Positive recombinant plasmids that contained an approximately 2.8-kb full-length TYLCV clone were fully sequenced using an ABI 3730XL by Macrogen (Seoul, Korea). Positive clones were named pTYLCV- [JU] and one positive clone was used in subsequent work.

### Construction of TYLCV infectious clone

An infectious TYLCV clone was constructed using a 1.8-mer genome-length copy of TYLCV obtained from pTYLCV- [JU]. For this purpose, a 2.35 kb *Eco*RI/*Nco*I fragment of pTYLCV- [JU] was first cloned into the pCAMBIA1380 binary plasmid, which had been digested with *Eco*RI and *Nco*I to create pBTY [JU]P. Then, a 2.8 kb *Nco*I fragment of pTYLCV- [JU], the full-length TYLCV clone, was ligated into an *Nco*I-linearized pBTY [JU]P yielding pBTY [JU], a binary plasmid containing a 1.8-mer of TYLCV- [JU].

### *In vitro *culture of plants

Seeds of the TYLCV- susceptible *S. lycopersicum *line "NS16" were obtained from an advanced breeding program developed at the Jordanian National Seed Company. The NS16 tomato line originates from a cross between *S. lycopersicum *cv. Guardian (developed by the Enza-Zaden Seed Company), a highly TYLCV-susceptible tomato and *S. lycopersicum *cv. Elegro (developed by the Asgrow Seed Company), a TYLCV-resistant tomato (N. Abu Al Roz, personal communication). NS16 is a determinate tomato plant that yields dark red fruits of 220-250 g. Seeds of *S. lycopersicum *cv. Moneymaker were obtained from Gourmet Seed International Seed Company, USA. The seeds of *S. habrochaites *accession LA 1777 were kindly provided by C.M. Rick, TGRC, Davis, USA.

Seeds from plants of NS16, Moneymaker and LA 1777 were surface sterilized with 70% ethanol for one minute followed by soaking for 15 minutes in 3.5% sodium hypochlorite plus 0.1% Tween-20. Seeds were rinsed 6 times with sterile water and then placed in a plastic vessel containing MS medium consisting of MS salt [20], 50 mg/l myo-insitol, 2 mg/l thiamine HC1, 0.5 mg/l pyridoxine HCl, 0.5 mg/l nicotinic acid, 30 g/l sucrose and 7 g/l agar, with pH value adjusted to 5.8 before autoclaving. Seeds were germinated in a growth room (24°C under cool white fluorescent lights, 50-100 μEm^-2 ^sec^-1^, with a photoperiod of 16 hours light/8 hours darkness). After 21 days, the tomato plantlets were used for the inoculation experiments.

### Inoculation methods

Competent cells of *Agrobacterium tumefaciens *strain GV3101 [21] were transformed by electroporation with either pCAMBIA1380 (negative control) or pBTY [JU]. Bacteria were grown for 24 hours at 28°C in Luria-Bertani (LB) media supplemented with appropriate antibiotics. Bacterial cells were harvested by centrifugation and resuspended to a final OD_600 _of 0.25 in liquid MS medium (without agar) supplemented with 100 μM acetosyringone. Into a sterile Petri dish, 10 ml of the bacterial suspension were poured. Microshoots (around 2.0 cm in length) were excised from 21 days old tomato plantlets and their basal parts were dipped for 30 seconds in the agrobacterial suspension. The inoculated microshoots (20 microshoot/inoculation test) were transferred into solid MS medium supplemented with 100 μM acetosyringone and co-cultivated with the bacteria for 48 hours. After 48 hours of co-cultivation, microshoots were washed three times in sterile distilled water containing filter-sterilized cefotaxim (500 mg/l) and then transferred to glass tubes containing 15 ml of solid MS medium supplemented with 500 mg/l cefotaxim, an antibiotic that kills bacterial cells. The inoculated microshoots were monitored for development of TYLCD symptoms for 8 weeks post-inoculation.

The effect of inoculum density on the TYLCV-infection efficiency was investigated using three different OD_600 _levels. For this purpose, agrobacterium cells were harvested and diluted to a final OD_600 _of 0.125, 0.25 or 0.5 in liquid MS medium supplemented with 100 μM acetosyringone and the bacteria were then co-cultivated with NS16 tomato microshoots and monitored for development of TYLCD symptoms.

The effect of the dipping method on TYLCV-infection efficiency was also tested. For this purpose, either the basal part of a NS16 tomato microshoot was dipped for 30 seconds or the entire microshoot was soaked for 15 minutes in the agrobacterium solution (OD_600 _of 0.25) and then washed three times in sterile distilled water containing filter-sterilized cefotaxim (500 mg/l). The explants were then co-cultivated and monitored for the development of TYLCD symptoms as described above.

To inoculate greenhouse grown tomato plants with TYLCV, a leaf agroinfiltration method was used. For this purpose, the bacterial suspension was prepared as described above and was then infiltrated into the lower side of leaves of 2-weeks old plants using a 1 ml needleless syringe. Inoculated plants were observed for the development of TYLCD symptoms for eight weeks post-inoculation.

### TYLCV detection in inoculated plants

To confirm the presence of TYLCV DNA in tissue-culture infected plants, PCR was performed using specific TYLCV primers. DNA extracts from the *in vitro*-inoculated plants were isolated as described by Doyle and Doyle [18]. The DNA extracts were used as template for PCR amplification (as described above) of a 450 bp long DNA fragment from the *C1 *gene using the primers TYMF (5'-AAGCGCTTCCAAATAAATTG-3') and TYMR (5'-TACTAATTCTTTAATGATTC-3'). The PCR products were subjected to electrophoresis in a 1% agarose gel.

To verify presence of the full-length circular single-stranded genome of TYLCV in inoculated plants, rolling circle amplification (RCA) [22] using bacteriophage *Phi29 *DNA polymerase (New England BioLabs, MA, USA) was carried out as follows: 1 μg of extracted total DNA was added to 5 μl of dNTPs (100 μM) and 5 μl random hexamers (2 pmol/μl). The mixture was heated to 95°C for 5 minutes, chilled on ice, and combined with 5 μl of reaction buffer plus 5 units of the *Phi29 *DNA polymerase. Water was added to a final volume of 50 μl. The reaction mixture was incubated for 18 h at 30°C, followed by inactivation of the enzyme at 65°C for 10 minutes. The amplified DNA, containing tandem repeats of the TYLCV genome, was separated in a 1% agarose gel. In addition, the amplified DNA was digested with *Nco*I in order to detect a ~2.8 kb band representing a linearized TYLCV genome in the inoculated plants.

## Results

### Establishment of the TYLCV *in vitro*-inoculation method

The complete genome of TYLCV was isolated from a tomato plant showing TYLCD symptoms using the overlapping primer method. The positive pTYLCV- [JU] clones were analyzed using several restriction enzymes and positive clones were completely sequenced. The isolate was found to show high sequence identity (99.4%) with the TYLCV-Mld isolate [23]. A 1.8-mer DNA copy from the isolated TYLCV genome was cloned into pCAMBIA1380 to produce the infectious clone pBTY [JU]. The infectious clone was tested successfully in greenhouse-grown plants of the TYLCV-susceptible tomato line "NS16" using the leaf agroinfiltration method (data not shown). Four weeks after leaf agroinfiltration with pBTY [JU], typical symptoms of TYLCD were observed in 13/15 of the inoculated plants, while tomato plants agroinfiltrated with pCAMBIA1380 remained symptomless.

To establish the TYLCV *in vitro*-inoculation method, basal parts of three weeks old microshoots obtained from *in vitro*-grown TYLCV-susceptible NS16 tomato plantlets were dipped for 30 seconds in solutions containing agrobacterium transformed with either the infectious clone pBTY [JU] or empty binary plasmid. The inoculated *in vitro *microshoots were monitored for development of TYLCV symptoms for 8 weeks post-inoculation. Table 1 summarizes the results obtained from three independent experiments. Over 85% of the *in vitro*-inoculated tomato microshoots with pBTY [JU] had typical TYLCD symptoms, while all of the tomato microshoots inoculated with the pCAMBIA1380 plasmid remained symptomless and continued their growth normally. However, 15% of the *in vitro*-inoculated tomato microshoots were considered healthy with no obvious TYLCD symptoms and they continued to grow normally (Table 1). The results obtained showed the feasibility to inoculate efficiently *in vitro *tomato plants with TYLCV and typical symptoms of TYLCD with leaf curling and yellowing were observed.

**Table 1:** Agroinoculation of *in vitro*-cultured NS16 and Moneymaker tomato plants with the infectious TYLCV clone pBTY [JU] and the negative control pCAMBIA1380.

Construct	Genotype	Experiment^a ^(% infected plants^b^)			
		
		I	II	III	Average
**pCAMBIA1380**	**NS16**	0 [0/20]	0 [0/20]	0 [0/20]	0 [0/60]
	**Moneymaker**	0 [0/20]	0 [0/20]	0 [0/20]	0 [0/60]
					
**pBTY [JU]**	**NS16**	85 [17/20]	80 [16/20]	90 [18/20]	85 [51/60]
	**Moneymaker**	95 [19/20]	100 [20/20]	95 [19/20]	96.7 [58/60]

^a ^In each experiment, 20 plants were inoculated.

^b ^Data were recorded 8 weeks post-inoculation. Infected plants percentages were determined from the numbers in brackets.

**Figure 1:**
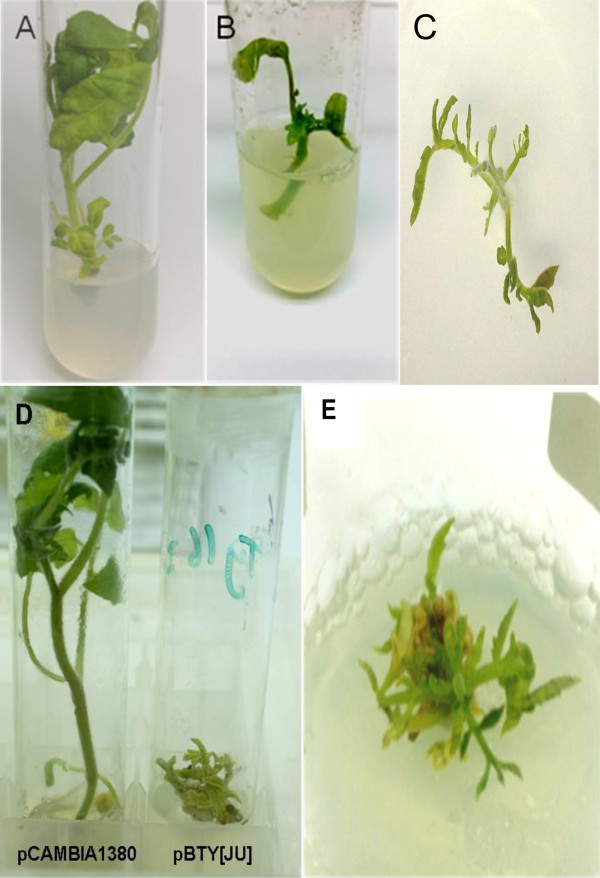
**Symptoms on *in vitro*-cultured NS16 tomato plants inoculated with the infectious TYLCV clone pBTY [JU]**. (A) Tomato plant 4 weeks after inoculation with pCAMBIA1380 (negative control). (B) Tomato plant 2 weeks after inoculation with pBTY [JU]. (C) Tomato plant 4 weeks after inoculation with pBTY [JU]. (D) Tomato plant 8 weeks after inoculation with pCAMBIA1380 (left) or pBTY [JU] (right). (E) Tomato plant 6 months after inoculation with pBTY [JU].

**Figure 2:**
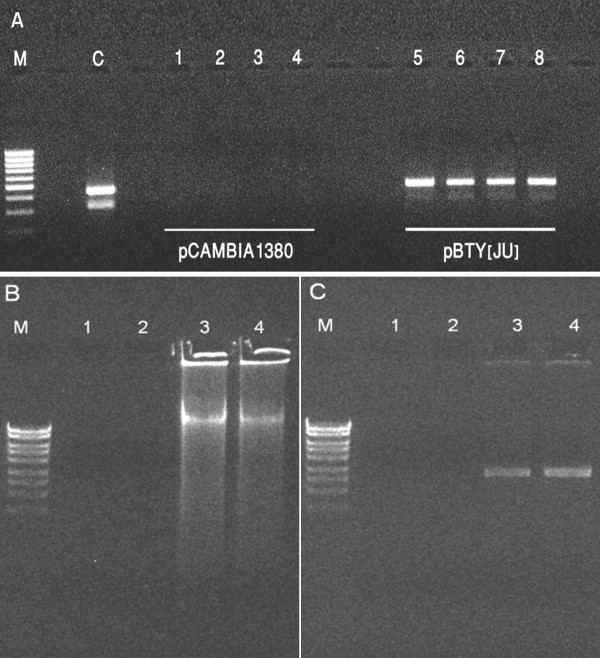
**Detection of TYLCV DNA in tissue-cultured NS16 tomato plants after inoculation with the infectious TYLCV clone pBTY [JU]**. (A) Agarose gel showing PCR products (450 bp) amplified with the primer pair TYMF/TYMR from DNA extracts of plants inoculated *in vitro *with TYLCV. Lanes 1-4: DNA extracts from plants inoculated with pCAMBIA1380 (negative control); Lanes 5-8: DNA extracts from plants inoculated with pBTY [JU]. C: pBTY [JU] plasmid (positive control). M: Low range DNA marker (Fermentas). (B) Agarose gel showing amplification products after rolling circle amplification (RCA) with DNA of plants inoculated with TYLCV *in vitro*. Lanes 1-2: DNA extracts from plants inoculated with pCAMBIA1380 (negative control); Lanes 3-4: DNA extracts from plants inoculated with pBTY [JU]. M: High range DNA marker (Fermentas). (C) Agarose gel showing TYLCV DNA after digestion of the RCA products with *Nco*I. Lanes 1-2: DNA extracts from plants inoculated with pCAMBIA1380 (negative control); Lanes 3-4: DNA extracts from plants inoculated with pBTY [JU]. M: High range DNA marker (Fermentas).

For symptom development, NS16 tomato microshoots inoculated with the empty plasmid did not show any TYLCD symptoms and continued to grow normally (Figure 1A). The development of TYLCD symptoms in some plants was first observed 2 weeks post inoculation (Figure 1B). After 4 weeks, NS16 tomato plants *in vitro*-inoculated with pBTY [JU] continued to display symptoms of stunting, upward leaf curling and yellowing (Figure 1C). After 8 weeks, the TYLCV-inoculated plants showed pronounced TYLCD symptoms when compared with plants inoculated with the pCAMBIA1380 plasmid (Figure 1D). The presence of TYLCV in the *in vitro*-inoculated plants showing TYLCD symptoms was verified by PCR and RCA (Figure 2). The TYLCD symptoms in *in vitro*-inoculated plants were similar to *in vitro*-cultured plants derived from nodal explants of tomato plants inoculated with TYLCV in the greenhouse (data not shown). The *in vitro*-inoculated plants with TYLCD symptoms were kept in culture for 6 months indicating the feasibility to maintain TYLCV *in vitro *using this method (Figure 1E). In addition, it was possible to transfer the *in vitro*-inoculated plants to greenhouse conditions after two weeks of acclimatization.

Furthermore, the *in vitro*-inoculation method was tested successfully and similar results were obtained with other TYLCV-susceptible tomato cultivars, such as Moneymaker (Table 1). The *in vitro*-inoculated Moneymaker plants showed similar TYLCD symptoms when compared to the *in vitro*-inoculated line NS16 microshoots after eight weeks of inoculation (data not shown). However, the *in vitro*-inoculated Moneymaker and "line NS16" plants showed variation in infection percentages (Table 1). In general, the *in vitro*-inoculated Moneymaker plants had higher infection percentage values when compared to the *in vitro*-inoculated line NS16 plants.

The effect of inoculum density on the TYLCV-infection efficiency was tested using three different OD_600 _(0.125, 0.25 or 0.5). The results from two independent experiments using NS16 tomato microshoots showed that bacterial densities of both 0.25 and 0.5 produced a high percentage of TYLCV infected plants (over 70% of inoculated plants) compared to a density of 0.125 (50% of inoculated plants) (Additional file 1). In some instances, plant death was observed (30% of inoculated plants) when an OD_600 _of 0.5 was used due to agrobacteria overgrowth (Additional file 1). These results indicate that an OD_600 _of 0.25 is considered optimum for inducing TYLCD symptoms in the *in vitro*-inoculation method.

**Figure 3:**
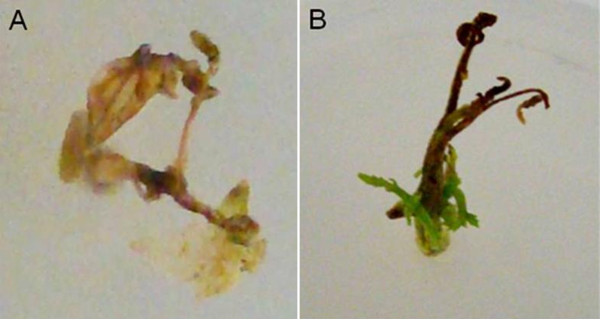
**Inoculation of *in vitro*-cultured NS16 tomato plants with the infectious TYLCV clone pBTY [JU] using the soaking method**. (A) Dead tomato plant 4 weeks after inoculation. (B) Growth of axillary bud in tomato plant 4 weeks after inoculation.

In the treatment where the whole plant was completely soaked in the agrobacterium solution, the percentage of TYLCV infectivity was very low compared to the treatment with 30 seconds of basal dipping (Additional file 2). The complete soaking of NS16 microshoots resulted in the browning and necrosis of all inoculated microshoots eight weeks post-inoculation (data not shown). In fact, 62.5% of plants soaked in the agrobacterium solution died four weeks post-inoculation (Figure 3A and Additional file 2). In addition, the surviving inoculated soaked plants (37.5% of inoculated plants) showed terminal bud death and the newly developed shoots from the axillary buds were infected and showed typical TYLCD symptoms (Figure 3B and Additional file 2). In some instances, the soaked plants showed overgrowth of agrobacteria four weeks post-inoculation, while with basal dipping no bacterial overgrowth was observed (data not shown). These results indicate that dipping the basal part of the explant is sufficient and suitable for the purpose of *in vitro*-inoculation of tomato plants with TYLCV.

### Testing the *in vitro *inoculation method with TYLCV-resistance plants

**Figure 4:**
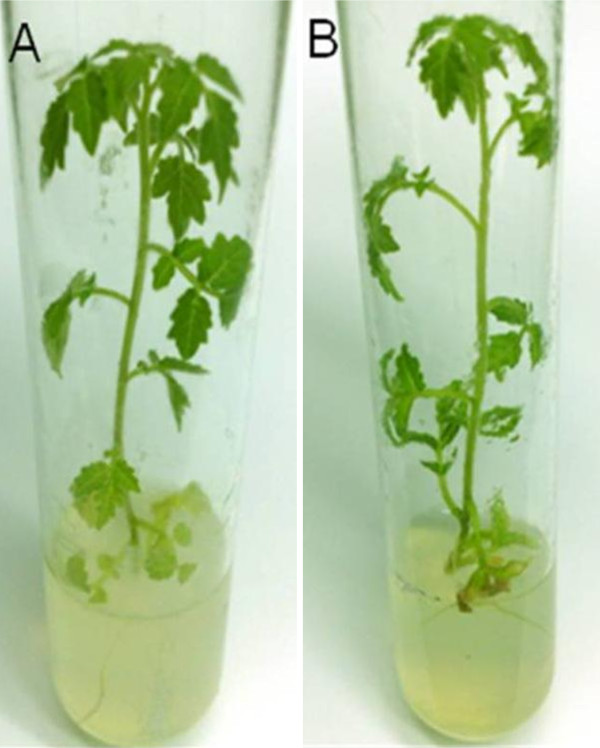
**Lack of symptoms for *in vitro*-cultured plants of *Solanum habrochaites *accession LA 1777 inoculated with the infectious TYLCV clone pBTY [JU]**. (A) Plant of *S. habrochaites *accession LA 1777 4 weeks after inoculation with pCAMBIA1380 (negative control). (B) Plant of *S. habrochaites *accession LA 1777 4 weeks after inoculation with pBTY [JU].

**Figure 5:**
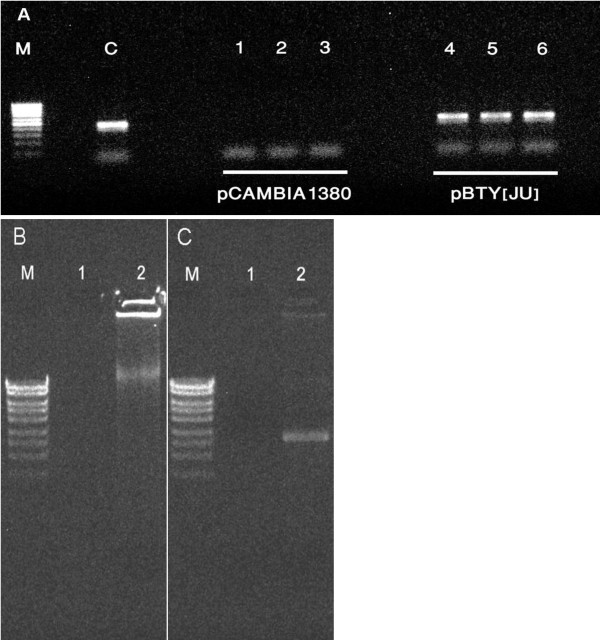
**Detection of TYLCV DNA in tissue culture plants of *Solanum habrochaites *accession LA 1777 after inoculation with the infectious TYLCV clone pBTY [JU]**. (A) Agarose gel showing PCR products (450 bp) amplified with the primer pair TYMF/TYMR from DNA extracts of plants inoculated *in vitro *with TYLCV. Lanes 1-3: DNA extracts from plants inoculated with pCAMBIA1380 (negative control); Lanes 4-6: DNA extracts from plants inoculated with pBTY [JU]. C: pBTY [JU] plasmid (positive control). M: Low range DNA marker (Fermentas). (B) Agarose gel showing amplification products after rolling circle amplification (RCA) with DNA of plants inoculated with TYLCV *in vitro*. Lane 1: DNA extract from plant inoculated with pCAMBIA1380 (negative control); Lane 2: DNA extract from plant inoculated with pBTY [JU]. M: High range DNA marker (Fermentas). (C) Agarose gel showing TYLCV DNA after digestion of the RCA products with *Nco*I. Lane 1: DNA extract from plant inoculated with pCAMBIA1380 (negative control); Lanes 2: DNA extracts from plants inoculated with pBTY [JU]. M: High range DNA marker (Fermentas).

To test the *in vitro*-inoculation method with TYLCV-resistant wild tomato plants, *in vitro *cultures of *S. habrochaites *accession LA 1777 were established and microshoots were inoculated and monitored for TYLCD symptoms as described above. *S. habrochaites *LA 1777 is known for its high levels of resistance against the whitefly insect, the transmission of the virus by the insect and its tolerance to TYLCV and it is commonly used in breeding programs to produce tomato plants with improved resistance against TYLCV [24]. In contrast to the results obtained with the *in vitro*-inoculation of susceptible tomato plants, all LA 1777 microshoots (results of two experiments with 20 microshoots per experiment) did not show any TYLCD symptoms even 4 weeks after inoculation and they continued to grow normally and similarly to microshoots inoculated with the empty binary plasmid (Figure 4). Although LA 1777 plants were symptomless, PCR and RCA analysis showed the presence of TYLCV DNA in inoculated plants verifying the presence of a resistance mechanism [23] against the virus preventing TYLCD symptoms development (Figure 5). These results indicate the possibility to identify the mechanism of resistance against TYLCV in wild tomato genotypes using *in vitro *cultures.

## Discussion

In this study, an efficient *in vitro*-inoculation method for TYLCV was developed that is suitable for screening different tomato genotypes for their responses to TYLCV. The presented *in vitro*-inoculation method proved to be efficient and reliable. Such method is needed to overcome pitfalls reported for other TYLCV inoculation methods [3,8]. For instance, previous reports discourage the use of whitefly-inoculation methods due to difficulties related to controlling infection pressure, the escape of some plants from the infection and the influence of environmental conditions on TYLCD symptom development [9,10]. Furthermore, the *in vitro*-inoculation method can be adapted to inoculate plant species considered to be "non-host" by the whiteflies [8,10]. Inoculation methods depending on stem or leaf agroinfiltration under greenhouse conditions require extra measurements to avoid external infection and are inefficient in some plants [11,12,14]. Discrepancy in results of TYLCV inoculation using particle bombardment has been shown previously. For instance, Morilla et al. [14] successfully inoculated tomato plants with DNA of TYLCV- [Alm] from Almeria, while Ramos et al. [15] were unsuccessful even though they used a similar approach with TYLCV- [CU] from Cuba and TYLCSV. Furthermore, the efficiency of TYLCV infectivity using particle bombardment differed with the plant species [13]. Testing the *in vitro*-inoculation system on two different genotypes revealed the presence of variation in infection percentages (Table 1). Such variation in infection rates might indicate variability in responses to TYLCV infection between the two genotypes.

The described *in vitro *system is suitable for *in vitro *storage of TYLCV-infected plant material (Figure 1E). With this respect, Pelah et al. [25] reported the establishment of callus cultures from TYLCV-infected tomato plants that were suitable for *in vitro *storage of TYLCV-infected callus up to 8 months. Similar tissue culture approaches were developed for the purpose of *Tobacco mosaic virus *(TMV) propagation in hairy root cultures of *Nicotiana benthamiana *where the hairy root cultures were directly inoculated by the addition of the virus to the culture medium [26]. Therefore, the described *in vitro*-inoculation method can be used for prolonged storage of infected material and can be used in exchanging infected plant materials between locations.

Two previous reports describe systems suitable for the inoculation of *in vitro*-grown plants with viruses. Mazier et al. [16] have described a simple and efficient system for *in vitro *inoculation of lettuce plants with *Lettuce mosaic virus *(LMV). The principle of their method relies on the mechanical inoculation of *in vitro*-grown lettuce plantlets using latex fingers dipped in sap extract from greenhouse-grown infected plants. In another study, nodal cuttings from *in vitro*-cultured potato, tomato and tobacco plants have been infected *in vitro *with *Potato virus Y *(PVY) using mechanical and grafting inoculation [17]. However, the PVY symptoms on the *in vitro*-infected plants were not as obvious as those observed on greenhouse-grown infected plants. Both systems are based on mechanical inoculation and this is not applicable for TYLCV, which cannot be transmitted by mechanical inoculation. The *in vitro*-inoculation method described here needs agrobacterium to deliver the infectious TYLCV clone into *in vitro*-grown tomato plants. Similar to our approach, agroinoculation of aseptically grown *N. benthamiana *plants for the purpose of virus-induced gene silencing has been successful and targeted genes were silenced one week post-inoculation [12].

Similar to the method developed by Russo & Salck [17], the reported method is suitable for initial screening of virus resistance in transgenic plants. It can reduce the time needed to evaluate the performance of transgenic plants and it is suitable for testing such plants under controlled environment and thus meeting the regulations for testing transgenic plants. In addition, the current described *in vitro*-inoculation method can be used to test the responses of different plant species to inoculation with TYLCV strains not prevalent in certain geographical areas. This would prevent the spread of viral strains to new areas, which is a risk issue when using inoculations in greenhouses or fields. The described method can facilitate studying the biological interactions between different tomato genotypes and different begomoviruses. In addition, the developed *in vitro*-inoculation method can be adapted to infect tomato plants simultaneously with different viral strains or species, which is difficult to perform using natural or whitefly-inoculation methods. Furthermore, the method is suitable for testing the specificity of interaction between different tomato genotypes and TYLCV strains avoiding cross contamination with other viruses and pathogens that are common using inoculations in greenhouses or fields. Additionally, the described *in vitro*-inoculation method is aseptic and it will eliminate the presence of other pathogens that might cause overlapping symptoms with the TYLCD *in vivo*.

The TYLCD symptoms were obvious on the *in vitro*-inoculated plants, although sometimes overlap with the tissue culture-induced phenotype was observed (Figure 1). However, the TYLCD symptoms of plants inoculated *in vitro *were similar to *in vitro*-cultured plants derived from nodal explants of tomato plants inoculated with TYLCV in the greenhouse (data not shown). The TYLCV symptoms were absent when the *in vitro*-inoculation method was tested on wild tomato plants known for their resistance against TYLCV. Therefore, it is necessary to detect the virus using molecular tools such as PCR, Southern blot analysis or RCA.

The *in vitro*-inoculation method described here can be used to understand the mechanisms of resistance against TYLCV in wild tomato genotypes. Using this method with *S. habrochaites *LA 1777, a wild tomato showing resistance to both whitefly and TYLCV [24], the presence of a TYLCV resistance mechanism was unmasked (Figure 4). Using the *in vitro*-inoculation method, the TYLCV-inoculated LA 1777 microshoots were symptomless for TYLCD, but tested positive for TYLCV using PCR and RCA (Figure 5). LA 1777 plants grown under greenhouse conditions and subjected to inoculation with viruliferous whiteflies and PCR analysis revealed the presence of both immune (virus is not detectable in the plant) and tolerant (virus is detectable in the plant, but the TYLCD symptoms are absent) mechanisms against TYLCV [unpublished results], which is consistent with previous reports [24]. Several attempts to transmit TYLCV to LA 1777 through grafting with infected tomato plants or natural infection under greenhouse conditions failed [unpublished results]. The current method can overcome such limitations related to incompatibility between scion and stock or natural inoculation difficulties due to whitefly non-preference. According to Vidavsky & Czosnek [24], the mechanisms of resistance in LA 1777 are expressed at the whitefly-plant interface (viral transmission) and internally in the plant (TYLCD symptoms development); therefore, by using natural inoculation methods, the resistance at the whitefly-plant interface will mask the resistance toward the virus inside the plant. Using the described *in vitro*-inoculation method, it was possible to overcome such limitation and it was feasible to uncover the natural resistance of LA 1777 to TYLCV. This is in general agreement with the results of Kheyr-Pour et al. [27], where *in vivo *agroinoculation was used to break the TYLCV resistance in LA 1777.

## Conclusions

In this study, an efficient method suitable for *in vitro *inoculation of tomato plants with TYLCV was developed. The method was used efficiently to unmask the TYLCV resistant in wild tomato. The current method allows the storage and propagation of infected tomato plants under proper controlled conditions. The described *in vitro *method will be recommended for initial screens of transgenic plants with improved resistance against TYLCV.

## Competing interests

The authors declare that they have no competing interests.

## Authors' contributions

AMA conceived the research, performed most of the experiments, and wrote the manuscript; HSD collected the samples and monitored the TYLCD symptoms development; SM helped in the molecular work; HA and AQ helped in the tissue culture work; AK developed the conceptual aspects of the work and edited the manuscript; All authors read and approved the final manuscript.

## Supplementary Material

Additional file 1 **Effect of inoculum density on *in vitro*-cultured NS16 tomato plants inoculated with the infectious TYLCV clone pBTY [JU]**. A table showing the responses of tomato microshoots following the inoculation with three different inoculum densities of agrobacteria harboring the infectious TYLCV clone pBTY[JU]. Click here for file

Additional file 2 **Effect of inoculation method on *in vitro*-cultured NS16 tomato plants inoculated with the infectious TYLCV clone**. A table showing the responses of tomato microshoots to the inoculation with the infectious TYLCV clone pBTY [JU] using two different methods. Click here for file
