# Supplementary material for: An efficient in vitro-inoculation method for Tomato yellow leaf curl virus
Source: Virol J. 2010 Apr 29;7:84. doi: 10.1186/1743-422X-7-84 (PMC2874538; doi:10.1186/1743-422X-7-84)
Supplement: Additional file 1 — Effect of inoculum density on in vitro-cultured NS16 tomato plants inoculated with the infectious TYLCV clone pBTY [JU]. A table showing the responses of tomato microshoots following the inoculation with three different inoculum densities of agrobacteria harboring the infectious TYLCV clone pBTY[JU]. [file 1743-422X-7-84-S1.DOC]

**Effect of inoculum density on *in vitro***-cultured NS16 tomato plants inoculated with the infectious TYLCV clone pBTY[JU].

| **Inoculum density (OD_600_)** | **Experiment I^a, b^** | | | **Experiment II** | | | **Average** | | |
| --- | --- | --- | --- | --- | --- | --- | --- | --- | --- |
|  | **Infected plants %^+^** | **Healthy plants %^++^** | **Dead plants %^+++^** | **Infected plants %** | **Healthy plants %** | **Dead plants %** | **Infected plants %** | **Healthy plants %** | **Dead plants %** |
| **0.125** | 45 [9/20] | 55 [11/20] | 0 [0/20] | 55 [11/20] | 45 [9/20] | 0 [0/20] | 50 [20/40] | 50 [20/40] | 0 [0/40] |
| **0.25** | 80 [16/20] | 20 [4/20] | 0 [0/20] | 85 [17/20] | 15 [3/20] | 0 [0/20] | 82.5 [33/40] | 17.5 [7/40] | 0 [0/40] |
| **0.5** | 70 [14/20] | 0 [0/20] | 30 [6/20] | 70 [14/20] | 0 [0/20] | 30 [6/20] | 70 [28/40] | 0 [0/40] | 30 [12/40] |

**^a^ In each experiment, 20 plants were inoculated.**

**^b^ Data were recorded 8 weeks post-inoculation; ^+^: Percentages of infected plants were determined from the numbers of plants showing TYLCD symptoms over the number of inoculated plants. ^++^: Percentages of healthy plants were determined from the numbers of plants without TYLCD symptoms over the number of inoculated plants. ^+++^: Percentages of dead plants were determined from the numbers of dead plants over the number of inoculated plants. Between brackets, number of plants in each category.**
