# Supplementary material for: An efficient in vitro-inoculation method for Tomato yellow leaf curl virus
Source: Virol J. 2010 Apr 29;7:84. doi: 10.1186/1743-422X-7-84 (PMC2874538; doi:10.1186/1743-422X-7-84)
Supplement: Additional file 2 — Effect of inoculation method on in vitro-cultured NS16 tomato plants inoculated with the infectious TYLCV clone. A table showing the responses of tomato microshoots to the inoculation with the infectious TYLCV clone pBTY [JU] using two different methods. [file 1743-422X-7-84-S2.DOC]

**Effect of inoculation method on *in vitro*-cultured NS16** tomato plants inoculated with the infectious TYLCV clone pBTY[JU].

| **Inoculation method** | **Experiment I^a, b^** | | | **Experiment II** | | | **Average** | | |
| --- | --- | --- | --- | --- | --- | --- | --- | --- | --- |
|  | **Infected plants %^+^** | **Healthy plants %^++^** | **Dead plants %^+++^** | **Infected plants %** | **Healthy plants %** | **Dead plants %** | **Infected plants %** | **Healthy plants %** | **Dead plants %** |
| **Basal dipping** | 80 [16/20] | 20 [4/20] | 0 [0/20] | 90 [18/20] | 10 [2/20] | 0 [0/20] | 85 [34/40] | 15 [6/40] | 0 [0/40] |
| **Soaking** | 40 [8/20] | 0 [0/20] | 60 [12/20] | 35 [7/20] | 0 [0/20] | 65 [13/20] | 37.5 [24/40] | 0 [0/40] | 62.5 [16/40] |

**^a^ In each experiment, 20 plants were inoculated.**

**^b^ Data were recorded 8 weeks post-inoculation; ^+^: Percentages of infected plants were determined from the numbers of plants showing TYLCD symptoms over the number of inoculated plants. ^++^: Percentages of healthy plants were determined from the numbers of plants without TYLCD symptoms over the number of inoculated plants. ^+++^: Percentages of dead plants were determined from the numbers of dead plants over the number of inoculated plants. Between brackets, number of plants in each category.**
